# X-rays are as effective as gamma-rays for the sterilization of *Glossina palpalis gambiensis* Vanderplank, 1911 (Diptera: Glossinidae) for use in the sterile insect technique

**DOI:** 10.1038/s41598-023-44479-8

**Published:** 2023-10-17

**Authors:** Bénéwendé Aristide Kaboré, Arooj Nawaj, Hamidou Maiga, Olga Soukia, Soumaïla Pagabeleguem, Marie Sophie Gisèle Ouédraogo/Sanon, Marc J. B. Vreysen, Robert L. Mach, Chantel J. de Beer

**Affiliations:** 1 Joint FAO/IAEA Centre of Nuclear Techniques in Food and Agriculture, International Atomic Energy Agency, Vienna International Centre, Vienna, Austria; 2 Institute of Chemical, Environmental and Bioscience Engineering, Vienna University of Technology, Vienna, Austria; 3 Insectarium de Bobo-Dioulasso-Campagne d’Eradication de la mouche Tsé-tsé et de la Trypanosomose, Bobo-Dioulasso, Burkina Faso; 4 Present Address: Institut de Recherche en Sciences de la Santé/Direction Régionale de l’Ouest (IRSS-DRO), Bobo-Dioulasso, Burkina Faso; 5 Present Address: Joint FAO/IAEA Centre of Nuclear Techniques in Food and Agriculture, International Atomic Energy Agency, Vienna International Centre, Vienna, Austria

**Keywords:** Animal biotechnology, Assay systems, Behavioural ecology

## Abstract

An area-wide integrated pest management strategy with a sterile insect technique (SIT) component requires a radiation source for the sterilisation of male insects. Self-contained gamma irradiators, which were exclusively used in past SIT programmes, are now facing increasing constraints and challenges due to stringent regulations. As a potential alternative, new generation high output X-ray irradiators have been proposed. The feasibility of using X-ray irradiators was assessed by comparing the effects of both gamma- and X-ray irradiators on biological parameters of *Glossina palpalis gambiensis* (Vanderplank, 1911), that are important for SIT applications. The gamma irradiator Foss Model 812 and two X-ray irradiators, the Rad Source 2400 and the blood irradiator Raycell Mk2 were used. *Glossina palpalis gambiensis* males were exposed to radiation as pupae. A radiation dose of 110 Gy or above induced more than 97% sterility in females that mated with the irradiated males for all the irradiators. Adult emergence rate, flight propensity, survival and mating performance did not differ between gamma- and X-rays irradiators. These results suggest that irradiating pupae with a dose of 110 Gy is optimal for both gamma-and X-ray irradiators used in this study, to achieve a sterility of approximately 99%. Similar research on other tsetse species could gradually phase out the use of gamma-ray irradiators in favour of X-rays irradiators, especially for smaller SIT programmes.

## Introduction

Insect pests that transmit pathogens and parasites to livestock are responsible for the loss of billions of dollars in agriculture and livestock production globally. Furthermore, climate change will likely contribute to a change in the traditional geographic distribution of insect-borne pathogens.

In Africa, tsetse flies (Diptera: Glossinidae) are the cyclical vectors of *Trypanosoma* spp. that cause Human African Trypanosomosis (HAT), known as sleeping sickness, and African Animal Trypanosomosis (AAT) known as nagana. Sleeping sickness caused devastating epidemics during the twentieth century and if left untreated, the disease can lead to death^1^. Nagana remains a neglected disease that causes a reduction in animal health and productivity^2,3^. As such, it represents a major obstacle to the expansion of livestock breeding and livestock-based industries in humid and sub-humid zones in 10 million km^2^ of sub-Saharan Africa^4^.

A variety of techniques have been developed to suppress or eradicate populations of tsetse flies. Although insecticide-impregnated targets/traps, live bait technologies and the sequential aerosol technique have been successful in clearing some areas containing tsetse flies, most of these areas have later been re-invaded as most of these programmes were not implemented on an area-wide basis^5^. Area Wide-Integrated Pest Management (AW-IPM) with a Sterile Insect Technique (SIT) component can potentially eliminate a population of a specific insect species within a circumscribed target area. In the 1930s, E.F. Knipling suggested that sterile males could be used to reduce or eradicate wild populations of specific pest insects^6,7^. This idea led to the largest and most successful AW-IPM programme, integrating a SIT component, implemented over a period of 50 years, i.e., the eradication of the New World screwworm *Cochliomyia hominivorax* (Coquerel, 1858) (Diptera: Calliphoridae) from the southern USA, Mexico, the rest of Central America and Panama^7,8^ . The SIT was successfully used, in combination with other control methods, to eradicate, suppress or contain several insect pest populations including the eradication of the pink bollworm *Pectinophora gossypiella* (Saunders, 1844) (Lepidoptera; Gelechiidae) in the southern USA and northern Mexico, the containment of the Mediterranean fruit fly *Ceratitis capitata* (Wiedemann, 1824) (Diptera; Tephritidae) in Guatemala and Mexico^9,10^ and suppression of the codling moth, *Cydia pomonella* (Linnaeus, 1758) (Lepidoptera; Tortricidae) in the Okanagan Valley of British Colombia, Canada^11^ and the suppression of *Ceratitis capitata* (Wiedemann, 1824) in South Africa, Israel and Jordan^12,13^. This technique was demonstrated on the Island of Unguja, Zanzibar in 1994–97 where a population of *Glossina austeni* (Newstead, 1912) was eradicated using a combination of control tactics, i.e., insecticide impregnated targets, pour-on treatments on livestock and SIT^2,14^. The same approach was successfully used against a population of *G. p. gambiensis* (Vanderplank, 1911) in the Niayes of Senegal^15^.

The SIT consists of the production of the target insect in large production facilities, the sterilisation of the male insects using ionizing radiation and the sustained and sequential release of the sterile insects in the target area^16^. The success of the SIT depends on the effective sterilization of the released male insects and the ability of these males to compete with their field counterparts for mating with wild females. Since the start of research on the development of the SIT package for the management of tsetse fly populations, the sterilization was mostly achieved using ionizing radiation^16,17^. Self-contained gamma irradiators that have a Cobalt-60 (^60^Co) source remain the most practical and extensively used radiation source in SIT programmes, whereas Cesium-137 (^137^Cs) sources have largely been phased out^18–20^. Gamma irradiators are, however, facing growing constraints and difficulties in terms of transport, import legislation, safety concerns and costs, limiting them only for programmes that produce large enough quantities of sterile insects^21–25^. As a result, some gamma irradiator manufacturers have been obliged to stop manufacturing these self-contained ^60^Co irradiators^23^. In the last two decades, efforts have been undertaken to assess whether a new generation of high output X-ray irradiators could likewise be used in these SIT programmes. As X-ray irradiators are not dependent on an isotopic source to produce radiation and their use is perceived as having fewer safety concerns, they have been proposed as an alternative to gamma irradiators in SIT programmes^23^. Regardless of the source of the radiation, the suitability of the type to be used for SIT programmes depends on properties such as relative biological effectiveness, penetrability, availability, safety and cost^26^. Before X-ray irradiators can be incorporated in SIT programmes, the feasibility to use these sources for insect sterilization needs to be determined and validated. Results obtained with mosquitoes, fruit flies and lastly the tsetse fly species *G. p. gambiensis* showed that X-ray irradiators can potentially be used for the sterilization of male insects of these target species and can serve as a cheaper and more practical alternative especially in smaller SIT programmes^20,23,24,27–29^. Indeed, X rays induced 99.8% of sterility in *Aedes aegypti* with doses of 55 Gy and above, 100% of sterility in *Anastrepha ludens* with 80 Gy, 99.9% in *Anopheles arabiensis* and *Ceratitis capitata* with 100 Gy, 99.6% in *G. p. gambiensis* at 110 Gy^30^. Compared to gamma-rays, X-rays induced comparable, and in some cases, slightly higher sterility levels in these fruit flies and human diseases vectors.

Following this recently demonstrated suitability of a blood X-ray unit for tsetse sterilization, the objective of the current study was to assess the dose responses of the target species *G. p. gambiensis* in the Niayes region of Senegal after exposure of pupae to radiation with two X-rays irradiators, in comparison with one gamma-ray irradiator. The biological parameters assessed included adult emergence rate, adult fly survival, induced sterility after mating of the treated males with non-irradiated females, flight propensity and mating performance or competitiveness. Our findings will supply the needed background to support the possibility of using X-ray sterilisation in the tsetse control programmes.

## Results

### Dosimetry

Gafchromic film was used to measure the absorbed dose. Supplementary Table S1. show the means, the 95% confidence interval, and the difference between the absorbed and targeted dose for the dose response evaluations, flight quality control and mating performance experiments. With the exception of the 130 Gy dose with the Foss Model 812 in the dose response evaluation (5.8%), the doses as measured with Gafchromic film did not differ significantly from the target doses at 5%. However, dose response curves were based on the absorbed dose.

### Experiment 1: dose response of pupae exposed to gamma- and X-rays

#### Emergence rate and female contamination

Adult emergence rate did not show significant differences based on the irradiators (χ^2^ = 2.5275; df = 1; p = 0.1119) or the radiation type (χ^2^ = 2.3864; df = 1; p = 0.1224). Furthermore, there were no significant effects on the emergence rate due to interactions between irradiators and doses (χ^2^ = 10.6905; df = 8; p = 0.2199). However, the radiation dose had a significant negative effect on the emergence rate (χ^2^ = 25.248; df = 4; p < 0.0001) which decreased with increasing radiation dose (Supplementary Fig. S1). The emergence rate was significantly higher in the control group as compared to the 110 Gy (p = 0.0218) and 130 Gy (p = 0.0002) treatment groups. Additionally, the emergence rate was significantly higher in the 70 Gy treatment group than the 130 Gy treatment group (p = 0.0014). Female contamination rate was 9.4 ± 4.7%. All females that emerged were not further used in the experiment and discarded.

#### Female and male survival

The survival curves of the non-irradiated females mated with the irradiated males are shown in Supplementary Fig. S2. The mean survival time was 57.1 ± 14.3 days. No statistically significant difference was observed in the survival time of flies between the irradiators (χ^2^ = 0.19; df = 2; p = 0.9085), nor the radiation type (χ^2^ = 0.126; df = 1; p = 0.7227) or the interaction between the irradiators and the radiation doses (χ^2^ = 3.3659; df = 8; p = 0.9093). The best model showed that only the radiation dose had a significant effect on survival (χ^2^ = 21.99; df = 4; p = 0.0002) (Supplementary Fig. S2). Females that mated with males irradiated as pupae with 70, 110 and 130 Gy survived significantly longer than females mated with non-irradiated males (p_70/0Gy_ = 0.0146; p_110/0 Gy_ = 0.0008; p_130/0 Gy_ = 0.0060). In the case of females that mated with males irradiated with 90 Gy, no statistically significant difference in lifespan was observed when compared to the control group (p = 0.3020).

**Figure 1:**
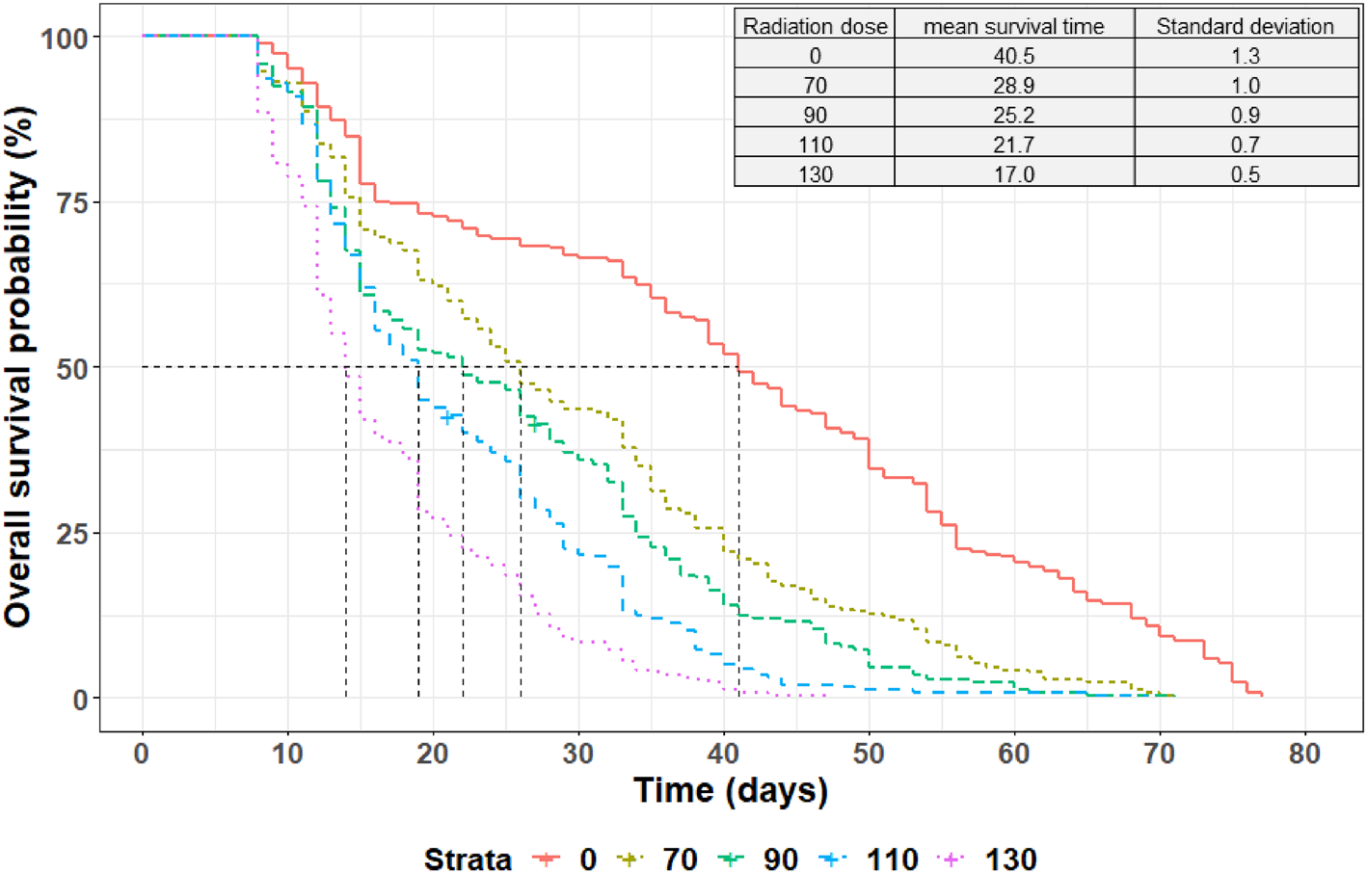
Survival curves of non-irradiated males *vs.* males irradiated with 70, 90, 110 and 130 Gy. The x-axis line shows the survival time in days and the black vertical lines indicate the median survival time (50% survival point) while the table display the mean survival time for each treatment.

The mean survival of the males under normal feeding conditions was 26.7 ± 16.7 days. No significant effect was observed in male survival probability between the irradiators (χ^2^ = 0.3439; df = 2; p = 0.842) or the radiation type (χ^2^ = 0.3052; df = 1; p = 0.5807). The interaction between the irradiators and the doses did not significantly affect the survival (χ^2^ = 5.2319 df = 8; p = 0.7325). The results of the best Coxme model showed that the radiation dose had a significant effect on the survival of the males (χ^2^ = 345.06; df = 4; p < 0.0001). In addition, survival decreased proportionally with increasing dose (Fig. 1).

#### Fecundity of females that mated with irradiated males

A total of 1770 non-irradiated virgin females were mated with irradiated and non-irradiated males and on day 18 (average age of first larviposition), 1658 were still alive, giving an overall survival rate of 93.7%. Supplementary Table S3 and Table S4 contain the adult emergence from irradiated pupae and the reproduction parameters of females mated with the irradiated and non-irradiated males. The three-parameter Weibull (W1.3) model was the best fitting model to describe the fecundity dose response curves. The graph was developed using the summarized data and predicted data from the experiments (Supplementary Fig. S3). Table 1 shows the results from the Weibull 3-parameter model. When comparing the three parameters i.e., upper limits, slopes and effective doses, no significant difference was found between the irradiators (Supplementary Table S2). Similar results were found when comparing the estimated effectives doses that reduced 50, 95 and 99% of fertility (p > 0.05).

**Table 1:** Weibull W1.3 model curves equations and the respective effectives doses that reduce fecundity of 50, 95 and 99% (95% CI).

Irradiator	Curve equation	D50 (95% CI)	D95 (95% CI)	D99 (99% CI)
Foss Model 812	y = 0.09exp (− exp(2.03(log(*x*) − 54.04)))	45.11 (31.31–58.92)	92.79 (81.21–104.38)	114.69 (87.54–141.84)
Rad Source 2400	y = 0.09exp(− exp(1.85(log(*x*) − 47.75)))	39.17 (22.98–55-35)	86.41 (75.82–96.99)	109.02 (81.54–136.49)
Raycell Mk2	y = 0.09exp (− exp(1.75(log(*x*) − 45.94)))	37.26 (22.30–52.24)	85.92 (75.42–96.42)	109.82 (82.74–136.90)
	compParm, p > 0.05	Kruskall-Wallis, χ^2^ = 2; df = 2; p = 0.3679		

The curves equations are expressed as y(x) = 0 + (d−0) exp(− exp(b(log(x) − e))) where the lower limit is fixed at 0, *d* is the upper limit, *b* is the slope and *e* is the effective dose; *x* represents the radiation dose needed to reduce a given fecundity.

**Figure 2:**
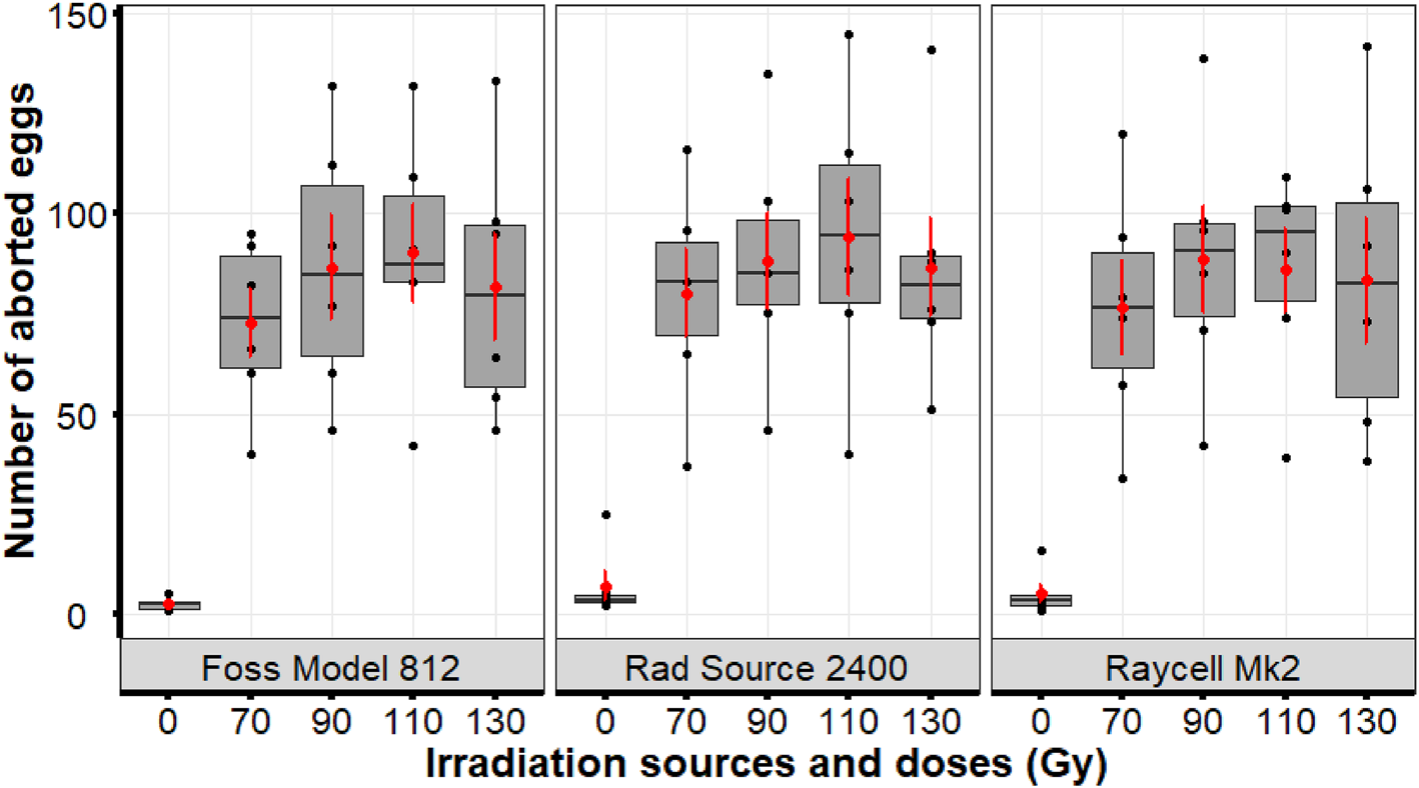
Number of ovulated eggs aborted during the 60-day experiment. Females that mated with males irradiated aborted a greater number of eggs as compared with those mated with non-irradiated males. The boxplot shows the median, and upper and lower quartiles while the means and the standard errors are shown in red.

The number of eggs aborted during the 60 days was similar in females mated with males irradiated with all the three irradiators (χ^2^ = 1.8823; df = 1; p = 0.1565).The GLMM with a negative binomial family showed that the number of eggs aborted varied significantly with dose (χ^2^ = 691.78; df = 4; p < 0.0001) while the irradiators had no significant effect (Fig. 2). The number of aborted eggs was higher in females mated with the irradiated males than females mated with non-irradiated males (p < 0.0001), and this was inversely related with pupae production (Supplementary Table S3). Additionally, the number of eggs aborted by females mated with males irradiated with 90 Gy and 110 Gy was higher (p_90Gy_ = 0.0177; p_110Gy_ = 0.0024) than those aborted by females mated with males irradiated at 70 Gy.

The emergence of the pupae produced by females mated with non-irradiated males was higher than those produced by females mated with irradiated males except those mated with males irradiated with 90 Gy in the Raycell Mk2 (95.8%) (Supplementary Table S3). The development time of flies in the produced pupae varied from 31 to 37 days. The male to female sex ratio of the emerged flies was about 1:1 (Supplementary Table S3).

Dissection results of surviving females on day 60 indicate that the insemination rate ranged from 0.99 to 1 for the females mated with non-irradiated males in comparison to a range from 0.91 to 0.99 for the females mated with irradiated males (Supplementary Table S4). Although the insemination rate was slightly higher in females mated with non-irradiated males than in those mated with irradiated males, radiation did not suppress the ability of males to transfer sperm, and there was no difference between the irradiators. The spermathecae fill score in females mated with non-irradiated as well as irradiated males was predominated by the score of 0.25, 0.50 and 0.75 for all irradiation doses and irradiators. Examination of the status of the uterus on day 60 in the dissected females showed a clear difference in its contents between females mated with non-irradiated males and females mated with irradiated males. The uteri of females that mated with males irradiated was mostly empty due to abortions or contained a recently ovulated egg in embryonic arrest. In contrast, the uteri of females that mated with non-irradiated males, contained recently ovulated eggs or viable instar larvae, while only a few were empty due to abortion (Supplementary Table S4).

#### Induced sterility

**Figure 3:**
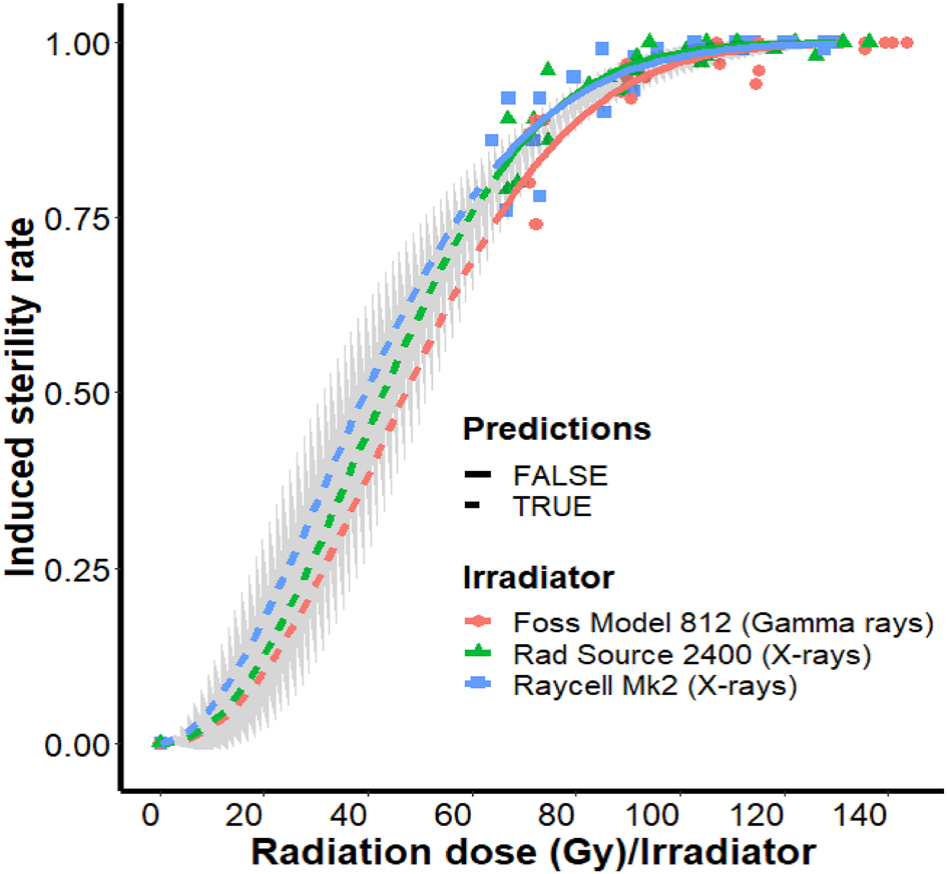
Weibull dose–response curves for the induced sterility in *Glossina palpalis gambiensis* pupae radiated with Foss Model 812 (Gamma rays), Rad Source 2400 (X-rays) and Raycell Mk2 (X-rays). The dashed lines indicate the predictive (Predictions = TRUE) irradiation doses and sterility while the solid lines represent the experimental data (Predictions = FALSE). The grey part indicates the 95% confidence interval.

Sterility induced in females mated with males irradiated as pupae averaged 82.2 ± 7.1% for the lowest dose of 70 Gy, while it averaged 99.8 ± 0.5% for 130 Gy and induced sterility increased with increasing dose (Fig. 3). Thus, 97.7%, 98.8% and 99.6% sterility were induced in the females mated with males irradiated with 110 Gy as pupae in the Foss Model 812, Rad Source 2400 and Raycell Mk2, respectively. Modelling the dose–response showed a good fit to the Weibull two parameters model (W2.2) (Table 2), and the model combining irradiators and doses was confirmed through the lack of fit test (F = 0.8724; Df = 57; p = 0.6749). There was no significant difference in the curves parameters between the irradiators as indicated in Supplementary Table S5. Specifically, the slopes of the curves and the effective median radiation doses were not significantly different between the irradiators (p > 0.05) (Supplementary Table S5). However, the parameter *e* was superior for the Foss Model 812 irradiator as compared with the Raycell Mk2 (p = 0.0834). This indicates that for achieving the same level of sterility, a lower irradiation dose is required for the Raycell Mk2 as compared with the Foss Model 812 irradiator. The estimated effective doses showed that the doses of Raycell Mk2 and Rad Source 2400 were lower (around 5 Gy less) than that of Foss Model 812. Indeed, the real data showed that Raycell Mk2 and Rad Source 2400 induced more than 95% sterility wi 90 Gy whereas a dose of 110 Gy Foss Model 812 was needed to reach the same level (Supplementary Table 3).

**Table 2:** Weibull W2.2 model curves equations and the respective effectives doses that induce 50, 95 and 99% of sterility (95%CI).

Irradiator	Curve equation	D50 (95% CI)	D95 (95% CI)	D99 (99% CI)
Foss Model 812	y = exp(− exp(2.17(log(*x*)−55.96)))	47.26 (39.75–54.78)	92.7 (86.06–99.44)	113.06 (98.06–128.07)
Rad Source 2400	y = exp(− exp(2.14(log(*x*)−51.09)))	43.06 (34.45–51.67)	85.24 (79.35–91.13)	104.18 (89.89–118.46)
Raycell Mk2	y = exp(− exp(1.86(log(*x*)−48.14)))	39.52 (31.7–47.27)	86.94 (81.03–92.85)	109.60 (95.16–124.04)
	compParm, p > 0.05	Kruskall-Wallis, χ^2^ = 2; df = 2; p = 0.3679		

The curves equations are expressed as y(x) = exp(− exp(b(log(x) − e))) where the lower limit was fixed at 0, *d* the upper limit was fixed at 1, *b* is the slope and* e* is the effective dose; *x* represents the radiation dose needed for a given induced sterility.

### Experiment 2: emergence rate, flight propensity, and survival

Mean adult emergence rate of the irradiated and control group pupae resulted in an overall average of 91.2 ± 5.5%. The statistical analysis showed that there was no significant difference in the emergence rate of irradiated pupae as compared with the control group (χ^2^ = 3.77; df = 3; p = 0.2878) (Supplementary Fig. S4). Prior to irradiation, all pupae were sex sorted using the Near Infra-Red Pupae Sex Sorter and the average female contamination observed was 5.25 ± 3.3%.

**Figure 4:**
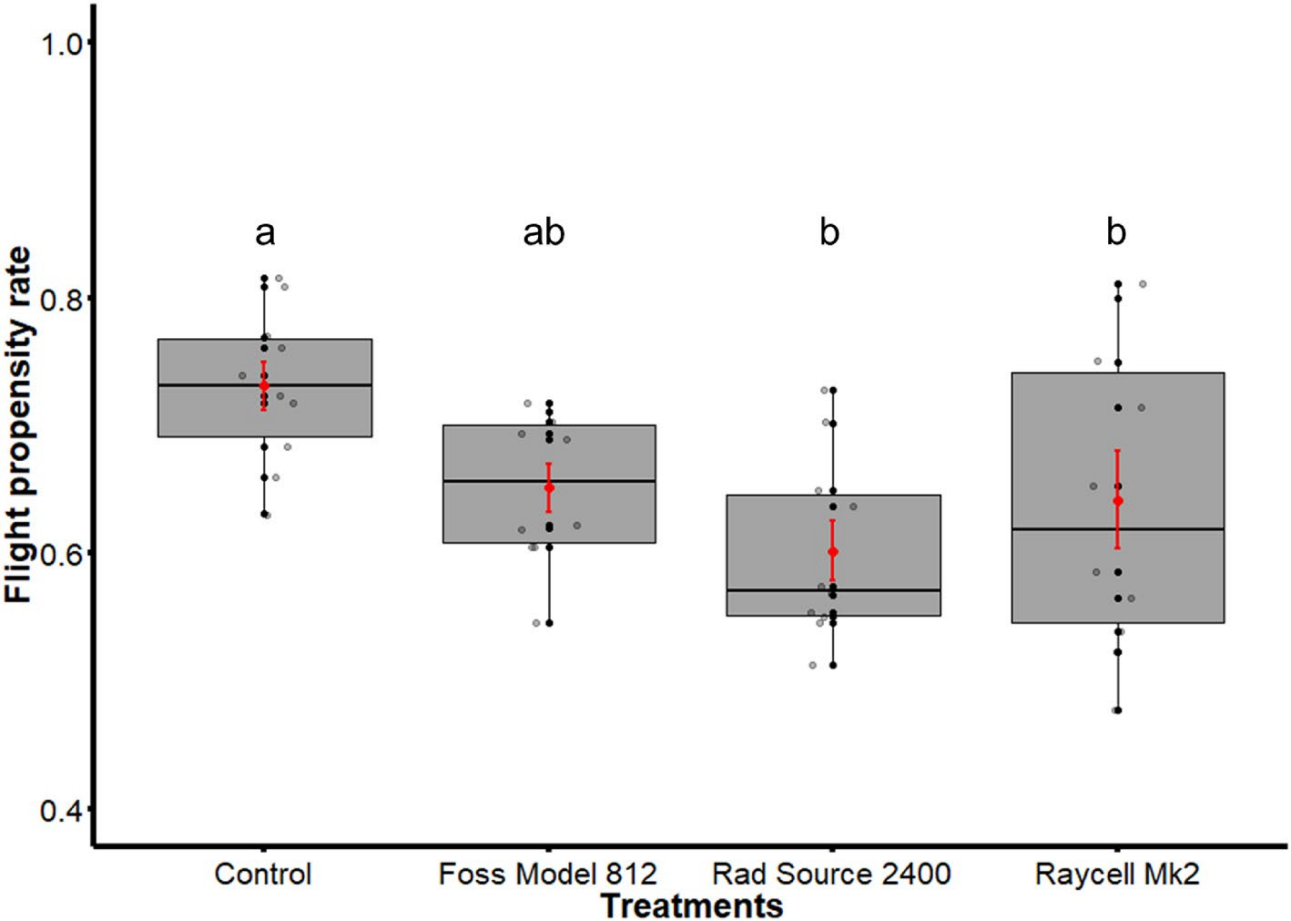
Flight propensity of males irradiated with the Foss Model 812, the Rad Source 2400 and the Raycell Mk2 *versus* non-irradiated males. The boxplot shows the median, upper and lower quartiles while the means and the standard errors are shown in red. Different letters indicate a significant difference between the treatments.

Flight propensity of males irradiated as pupae compared to those of the control group varied significantly (χ^2^ = 16.243; df = 3; p = 0.0010). Indeed, the flight propensity of the control group was significantly higher than those irradiated with both the Raycell Mk2 (p = 0.0242) and the Rad Source 2400 (p = 0.0005) while there was no significant difference with those irradiated with Foss Model 812 (p = 0.0568). No significant difference was detected among the irradiators (Fig. 4).

**Figure 5:**
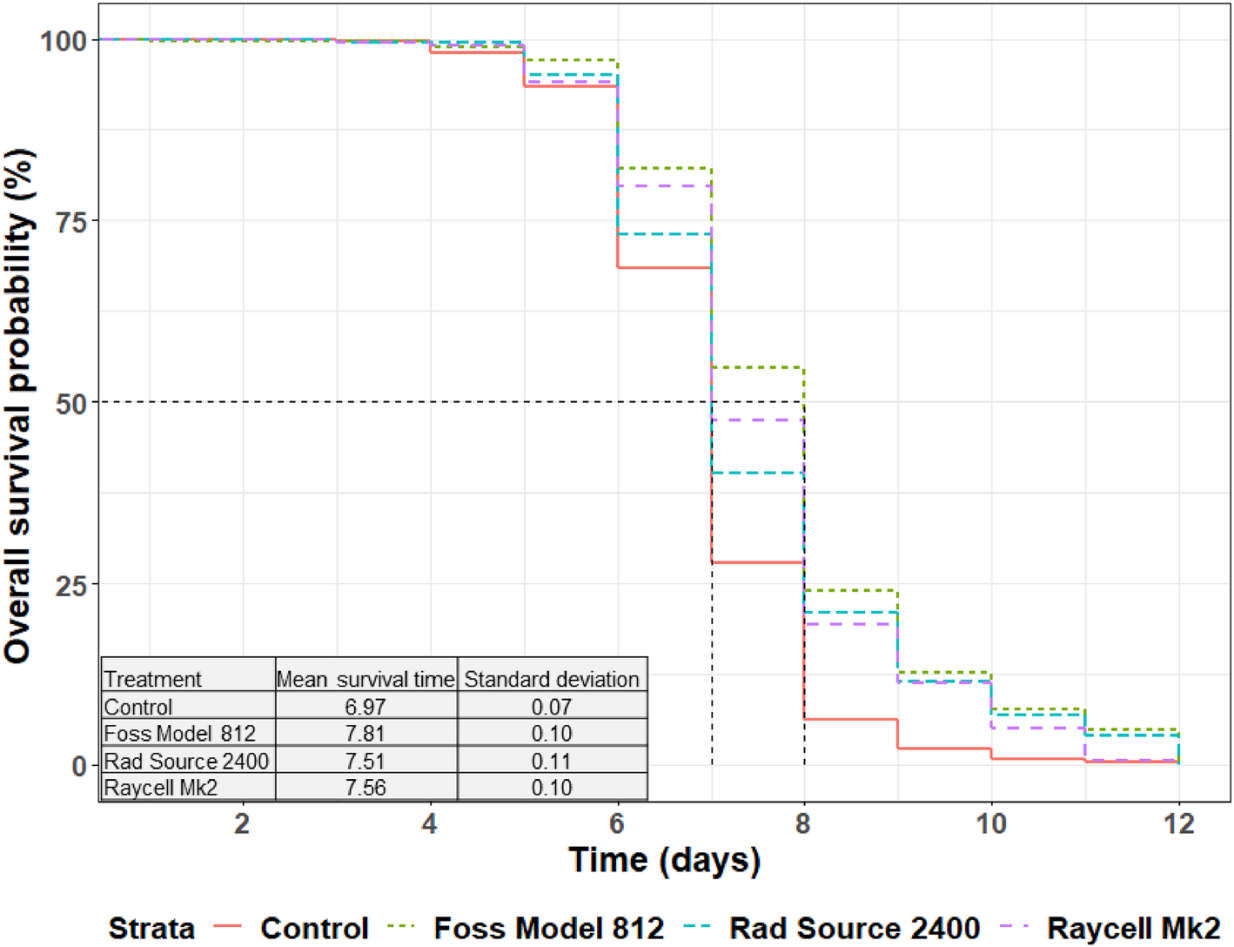
Survival curves of males irradiated with the Foss Model 812, the Rad Source 2400 and the Raycell Mk2 *versus* non-irradiated males. The x-axis line shows the survival time in days and the black vertical lines indicate the median survival time (50% survival point) while the table display the mean survival time for each treatment.

The survival time of males under feeding stress was significantly different between the treatments, i.e., between the irradiated males and the control group (χ^2^ = 47.55; df = 3; p < 0.0001). When computing a pairwise comparison, there was no significant difference between the survival time of the males irradiated with Foss Model 812 and both Rad Source 2400 (p = 0.1463) and Raycell Mk2 (p = 0.2916), nor between the X-rays irradiators (p = 0.9939). (Supplementary Table S6). Irradiated males from all the irradiators survived significantly longer than the non-irradiated males (Fig. 5).

### Experiment 3: male mating performance

#### Environmental conditions and fly behaviour in the field cage

The temperature during this assessment ranged from 21.1 to 27.5 °C, with the mean being 24.5 ± 1.5 °C. The relative humidity varied from 48.8 to 88.9%, with the mean being 71.1 ± 8.7%. Light intensity ranged from 112 Lux at the bottom of the cage to 2520 Lux at the top of the cage, with averages of 780.3 ± 330.0 Lux, 852.1 ± 449.8 Lux and 1357.3 ± 474.7 Lux at the bottom, mid/foliage and top of the cage, respectively. After the females were released, most flies aggregated at the top of the cage. Similar to the females, most of the released males also aggregated in the upper part of the cage, and immediate matings were usually observed. Subsequently, 81.8% of the mating pairs were collected at the upper half of the cage, where the average light intensity was 1239.83 ± 551.18 Lux (Supplementary Table S7).

#### Overall mating, relative mating index (RMI) and relative mating performance (RMP)

**Figure 6:**
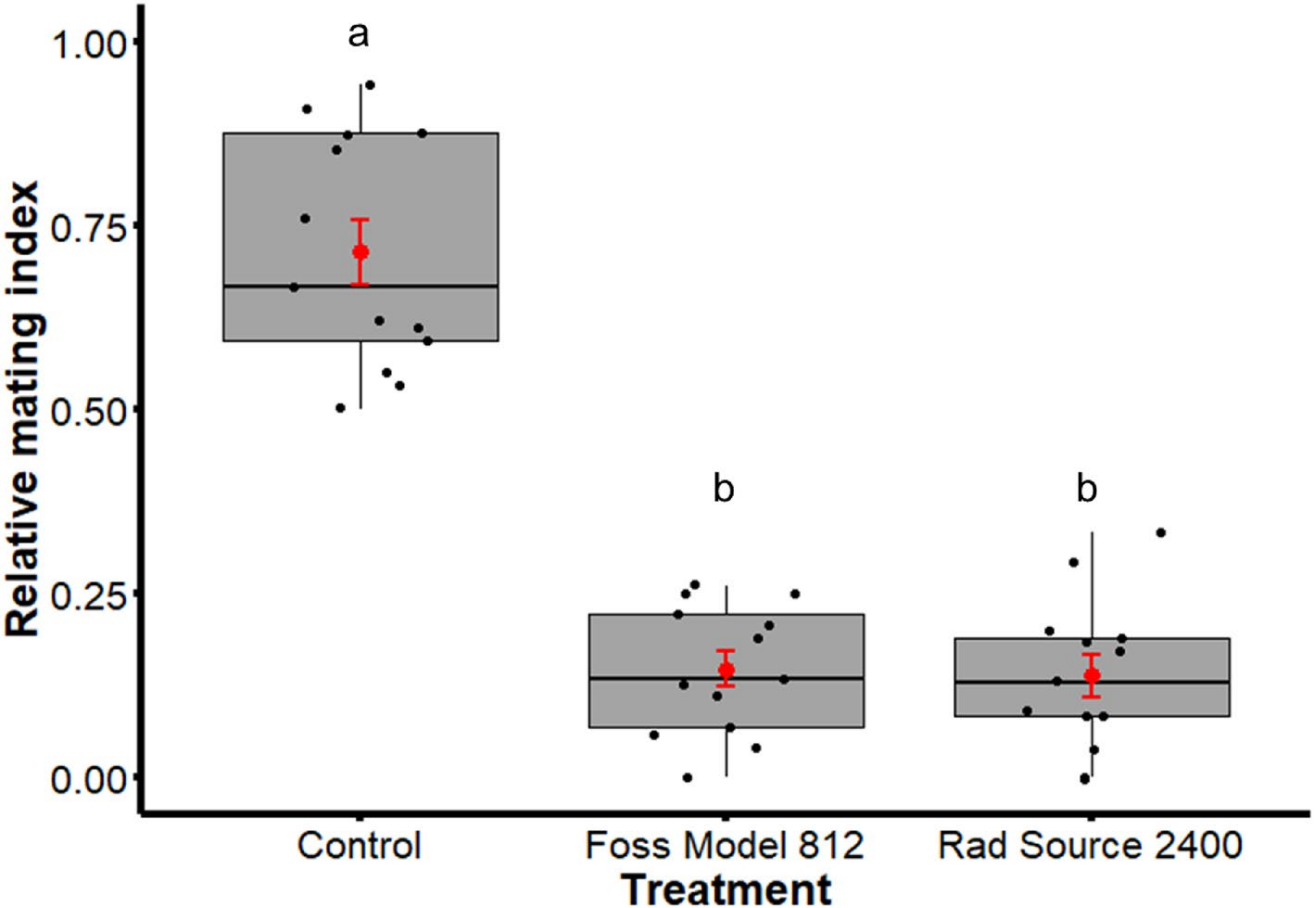
Comparison of the relative mating index (RMI) between non-irradiated males and males irradiated with Foss Model 812 and Rad Source 2400. The boxplot indicates the median, upper and lower quartiles while the means and the standard errors are highlighted in red. Different letters indicate a significant difference between the treatments.

Out of 390 possible pairs, 297 mating couples were formed, giving an overall propensity of mating (overall proportion of released females that mated) of 0.76 (Table 3). The RMI in Table 3 showed that radiation significantly decreased the mating ability of the males (χ^2^ = 87.32; df = 2; p < 0.0001). The RMI of the non-irradiated males was significantly higher as compared to the RMI of the males irradiated with the Foss Model 812 (p < 0.0001) and the Rad Source 2400 (p < 0.0001). No significant difference was observed between the two irradiators (p = 0.8634) (Fig. 6).

**Table 3:** Mean (± SD) of the main parameters of the mating performance experiment.

Treatment	Possible pairs	Actual mated	Overall proportion (PM)	Relative Mating Index	Relative mating performance	Mating latency (min)	Mating duration (min)	Spermathecae value (Mean ± SD)	Insemination rate
Control	–	213	–	71.39 ± 15.94^a^	–	35.39 ± 46.92^a^	86.59 ± 34.15^a^	0.88 ± 0.20^a^	0.99 ± 0.03^a^
Foss Model 812	–	44	–	14.78 ± 8.85^b^	− 0.57 ± 0.24	70.82 ± 59.43^b^	69.30 ± 36.41^b^	0.82 ± 0.26^ab^	0.97 ± 0.07^a^
Rad Source 2400	–	40	–	13.83 ± 10.34^b^	**− **0.58 ± 0.25	58.68 ± 51.97^b^	58.85 ± 30.84^b^	0.78 ± 0.27^b^	0.89 ± 0.28^a^
Global	390	297	0.76	–	–	43.77 ± 51.34	80.29 ± 35.52	0.86 ± 0.22	0.95 ± 0.17

Superscript letters indicate the p-values and different letters indicate a significant difference between the treatments.

The RMP defined as the difference between the numbers of mating pairs of irradiated males to non-irradiated males as a proportion of the total number of mating pairs, was − 0.57 ± 0.24 for the males irradiated with the Foss Model 812 as compared with − 0.58 ± 0.25 for those irradiated with Rad Source 2400 (Table 3).

#### Mating latency and duration

Mating latency (χ^2^ = 26.71; df = 2; p < 0.0001) and mating duration (χ^2^ = 38.96; df = 2; p < 0.0001) varied significantly between the treatments. Non-irradiated males and irradiated males were released simultaneously, and non-irradiated males were significantly faster to engage in mating as compared with those irradiated with the Foss Model 812 (p < 0.0001) and the Rad Source 2400 (p = 0.0034). However, there was no significant difference between the mating latency of the males irradiated with both irradiators (Supplementary Fig. 5). The couples formed by the non-irradiated males mated longer than the couples formed by males irradiated with the Foss Model 812 (p = 0.0009) and the Rad Source 2400 (p < 0.0001). However, the mating duration of males irradiated with both irradiators did not differ significantly (p = 0.6948) (Supplementary Fig. S6).

#### Insemination rate and spermathecae fill

Despite the significant difference in mating latency and duration between non-irradiated and irradiated males, no significant difference (χ^2^ = 1.33; df = 2; p = 0.2770) in insemination rate was observed. Among the treatments (irradiation vs control), mating duration, and their interaction in the linear model used to explain the spermathecae fill, only the treatment had a significant effect (χ^2^ = 7.3139; df = 2; p = 0.0258). However, using the pairwise comparison with adjusted p-values revealed that there was no significant difference between the treatments (Supplementary Table S8 and Table S9).

## Discussion

The established use of gamma irradiation for tsetse sterilization has prompted a search for alternatives, with X-rays, which, like gamma rays, induce DNA damage in insects through chromosome breaks, demonstrating high effectiveness in tsetse fly sterilization^30^. In the present study, the effectivity of a range of radiation doses (70–130 Gy) generated by two X-ray irradiators (Rad Source 2400 and Raycell Mk2) was compared with that of the same range generated by a gamma irradiator (Foss Model 812) for the sterilization of *G. p. gambiensis* pupae.

Adult male flies, that were irradiated as pupae with 110 Gy of gamma- and X-ray irradiators, induced more than 97% sterility in non-irradiated females. In addition, induced sterility increased as irradiation dose increased independent of the irradiator. The 97% sterility was higher than the 93.4% sterility obtained when adults of the same species were irradiated with the same dose of gamma-rays five decades ago^31^. Given the limitations of separating the sexes during the pupal stage at that time, Tazé et al.^31^ employed biological material consisting of adult males identified post-emergence. These adult males are recognized for their reduced sensitivity to irradiation compared to the pupae utilized in our experiment. The difference in sensitivity to radiation between tsetse development stages has been demonstrated in *G*. *brevipalpis* (Newstead, 1911)^32^ and *G. p. palpalis* (Robineau-Desvoidy, 1830)^33^, where pupae were found to be more sensitive to the radiation than adult flies.

It was also higher than the 89.7% sterility obtained in a recent study that used pupae of *G. p. gambiensis*^34^.

The pupae in the study of Ilboudo et al.^34^ were “under-dosed” as dosimetry revealed an absorbed dose of 81 Gy instead of 110 Gy. Furthermore, the absence of dosimetry and the potential for under-dosing, coupled with the protective effects of chilling^35^ could provide an explanation for the 120 Gy suggested by Pagabeleguem et al.^36^ when a re-evaluation of the dose response of male *G. p. gambiensis* has been done. These observations accentuate the importance of the implementation of an accurate and reliable dosimetry system in SIT facilities, as radiation dose is central to most radiobiological work^37^.

More than 95% sterility was induced in species such as *G. brevipalpis*^32,38^*, G. austeni* (Newstead, 1912)^39^, and *G. fuscipes fuscipes* (Newstead, 1911)^38^ with respective doses of 40 Gy, 80 Gy and 80–100 Gy. These doses differ from our finding of 110 Gy for *G. p. gambiensisis* (Vanderplank, 1911)*,* indicating species-specific variations in radiosensitivity. Therefore, the optimal dose in SIT programmes needs to be specified for each species, revisited periodically, and it remains to be seen whether the same range of radiosensitivity will be observed with X-ray irradiation. In addition to endogenous factors of insects that affect their radio-sensitivity, exogenous factors, such as handling, oxygen level, ambient temperature, dose-rate, and many others before and during irradiation, could influence the radio-sensitivity^40^.

The results of our study indicate that gamma- and X-ray irradiators induced similar sterility when *G. p. gambiensis* were irradiated as pupae, which suggests that X-ray irradiation can be an acceptable alternative to gamma irradiation. This finding is in agreement with a study on the navel orange worm which concluded that X- and gamma-rays treatments were biologically equivalent at similar doses^41^. These observations are dditionally supported by studies that show that X-ray irradiation can induce an acceptable level of sterility in several insects, such as mosquitoes^27,28,42^, Lepidoptera^43^ and fruit flies^20,41,44^.

In the current study, a dose of 90 Gy was sufficient to induce more than 95% sterility in females that had mated with males irradiated with one of the X-ray irradiators. This efficiency of X-rays might be related to their characteristics and the different dose rate of X-ray machines as compared with the Foss Model 812, since DNA damage due to radiation in multicellular organisms is dependent on the environmental dose rate^45^. Concerning the characteristics, gamma- and X-rays are waves in the electromagnetic spectrum, and gamma-rays have the shortest wavelength that are typically, but not always, shorter than those of X-rays (range from 10 pico- to 10 nm). These rays can be differentiated by their origin, i.e., gamma-rays are produced during nuclear decay of the nuclei of atoms, whereas X-rays are produced by electrons. Gamma-rays have a stronger ionizing ability. X-rays have less penetrating power as compared with gamma-rays (https://pediaa.com/difference-between-x-rays-and-gamma-rays/) and are widely used in the medical field. Secondly, in the current study, the dose rate of the Foss Model 812 was higher (between 76.4 and 68.9 Gy/min) than the dose rates of the X-ray irradiators (14.1 ± 0.7 Gy/min and 8.23 Gy/min for the Rad Source 2400 and the Raycell Mk2, respectively). The available literature on the impact of dose rate on the biological responses of insects shows that opinions are divergent. While several studies conclude that dose rate is a negligible parameter^46^, some showed that dose rate could have an influence on dose response of the insects, i.e., by increasing biological damage with increasing dose-rate^47–49^. A recent study, however, on mosquitoes showed that a low dose rate can achieve greater sterility than a high dose rate at high doses, while the inverse is seen in lower doses^49^.

Dissection results to determine uterus content in relation to ovarian development can be used to assess the effectiveness of sterile males in tsetse SIT programmes^50^. Similar to the results obtained with gamma-rays, females that mated with males subjected to X-rays as pupae had eggs in embryonic arrest in their uterus or showed an empty uterus probably due to the abortion of an embryo or larvae. The present results, independent of the irradiators, are in agreement with that obtained with *G. brevipalpis*^32^ and *G. austeni*^39^ following gamma radiation. The detectable imbalance between intra-uterine content and ovarian development reflects that there are higher proportions of dominant lethal mutations in the sperm of the males^38^ as the radiation dose increases.

Females mated with irradiated males (either gamma- or X-rays) survived longer than those mated with non-irradiated males, and this may be a result of their reproductive status. Lipolysis is required for milk production during tsetse pregnancy and is an indispensable and energy consuming process for the females mated with non-irradiated males.Unlike other insects, female tsetse flies undergo viviparous reproduction, producing a single instar larva in the uterus at any time, which is nourished by milk produced by the female's milk gland^51^. The absence of lactation and the stressful pregnancy cycle in females mated with irradiated males is the main reason for the prolonged female lifespan^52,53^. This result is of particular importance in tsetse control programmes and shows how accurate the sex-sorting system must be to eliminate the females from the release batches, as a longer life span implies more blood meals taken from host animals with a potential risk of transmission of trypanosome species by the released flies.

The current study shows that the survival of males that were offered blood meals decreased with increasing doses irrespectively whether gamma- or X-ray irradiators were used. In addition to dominant lethal mutations obtained in the sperm of males, irradiation causes undesirable somatic damage that is expressed as the development of abnormalities including a reduction in lifespan^26^.

Even though irradiating male pupae with 110 and 130 Gy induced more than 97% sterility in the non-irradiated females, the ultimate selection of the radiation dose for SIT programmes will depend on the competitiveness of the irradiated males^54,55^. Assessing flight quality and mating performance is therefore of particular importance. Based on the results of the dose–response curves generated in the current study, a dose of 110 Gy was selected to assess its effects on adult emergence, flight propensity, and male mating performance in a walk-in field cage.

Irradiation with either gamma- or X-ray irradiators showed that irradiation dose, regardless of the irradiator, had no significant negative effect on adult emergence. This agrees with results obtained in studies with the same species and the same radiation dose using gamma radiation^34,56^. As expected, with the exception of poor rearing conditions, only excessive irradiation doses or inappropriate handling of pupae may also reduce the emergence rate^57^. Flight propensity did not differ significantly between males irradiated with gamma- or X-rays irradiators, nor between those irradiated with the two X-ray irradiators. This result strengthens the hypothesis that at similar doses, the gamma- and X-rays have similar biological/physiological effects^41^.

Survival of irradiated males with gamma- and X-ray irradiators under stress was higher than the survival of non-irradiated males. Similar results were obtained with *G. pallidipes*^58^. The opposite was observed in a recent study^34^ although the median survival in our study (7 days for non-irradiated and irradiated flies respectively) was almost double of the one observed in the study of Ilboudo et al. (4.75 and 4.55 days for non-irradiated and irradiated males, respectively). The difference could be due to the sex-separation methodology of irradiated pupae that were sorted in the pupal stage with the NIRPSS in our study in comparison to chilled-sorting after adult emergence in the other one. In the absence of feeding, survival seems to be an inverse function of the body’s use of energy. It was observed that the irradiated males were less active and more lethargic as compared with the control group, the latter being very mobile and consequently using more energy. This difference in activity between irradiated and non-irradiated males could explain the longer survival of irradiated males. While these laboratory results could be perceived as a potentially negative consequence that may reduce mating or mate-seeking in the wild, the presence of various wild animals as feeding hosts could counterbalance this effect, increasing the likelihood of encounters with wild females and competition with wild male counterparts^59,60^. Indeed, when monitoring the experiment on survival under starvation, the irradiated starved flies attempt to bite the operator, suggesting that in the field, they will be active in the presence of hosts.

Mating performance in relation to the level of induced sterility is critical in the evaluation of the produced sterile males. As with several other parameters, none of the parameters characterising mating performance varied significantly between gamma- and X-ray irradiators except the average spermathecae fill. The overall mating propensity of 0.76 in the current study was higher than the one found in previous experiments with the same species and with the same irradiation target dose (0.57)^34^, and similar non-irradiated males (0.64)^61^. On the contrary, the PM was smaller than that of 0.83 found with non-irradiated males of *G. p. gambiensis*^62^. Other results indicated an overall propensity of 0.57 with *G. brevipalpis*^32^, while 0.63 and 0.41 were obtained with *G. austeni*^39^. These dissimilarities may be a result of a difference in treatments and environmental conditions as fly behaviour is indeed influenced by these^63^. However, the relative mating index indicates that the irradiated males are still competitive, and the small deficit as compared with non-irradiated male flies can be mitigated in SIT programmes by an increase in the sterile to wild male ratio. In addition, the insemination rate and spermathecal fill of females mated with males irradiated as pupae with gamma- and X-rays irradiators and with non-irradiated males were similar, as found in previous studies with *G. p. gambiensis, G. brevipalpis, and G. austeni*. These results confirm that irradiation does not affect their reproductive competence in the field^31,32,34,39^.

As males irradiated with X-rays can compete and inseminate similarly to males irradiated with gamma-rays and non-irradiated males under semi-field cage conditions, the use of X-rays seems to have potential for tsetse SIT programmes. However, treatment capacities are very relevant. Yamada et al.^30^ showed that it is possible to irradiate 1.1 million tsetse pupae in 5 days and two 6-h shifts per day using the Raycell Mk2 irradiator with 4 boxes of 1,500 pupae that can fit in the canister. Considering the same parameters, 35 boxes could fit in the five Rad Source 2400 canisters, while the Foss Model 812 chamber can hold 7 boxes. Considering the dose rates, the Foss Model 812 treatment capacity would be 7 times that of the Raycell Mk2, whereas it is almost equal to that of the Rad Source 2400 (1.2 times), the Rad Source 2400 capacity being 6 times that of the Raycell Mk2.

These results show that X-ray irradiators are well suited for tsetse pupae irradiation in large programmes, such as the one at “Insectarium de Bobo-Dioulasso-Campagne d’Eradication de la mouche Tsetse et de la Trypanosomose (IBD-CETT)”, which in 2022 shipped an average of 56,000 pupae per week to the Senegal eradication project in the Niayes (http://projet-tsetse-niayes.cirad.fr/). The highest number of sterile male tsetse released in one week was 102,557 during the successful SIT programme against *G. austeni* on Unguja Island (Zanzibar). Despite different treatment capabilities, X-ray irradiators are available and suitable for all sizes of SIT programme for irradiating pupae, unless bacterial decontamination of the blood by irradiation is considered^64^. In view of the constant low dose rate of X-ray irradiators, about 60 L and 600 L of blood could be irradiated with the Raycell Mk2 and the Rad Source 2400, respectively, in a 5-day week and two 6-h shifts daily, while the Foss Model 812 could irradiate about 900 L in the same period of time. In practical terms, the CIRDES and SEIBERDORSF strains of *G. p. gambiensis* in the IBD-CETT colony had an average size of ~ 500,000 females in 2022 and required 186 L of blood per week for feeding three times per week (IBD-CETT, unpublished data). Hence, X-ray irradiators could be used for blood irradiation, the Rad Source 2400 being more appropriate. While a single Rad Source 2400 unit is suitable for both blood and pupae irradiation in a programme of this size, four (04) Raycell Mk2 units with 2 L canister or two of 4.8 L canister option (http://www.theratronics.ca/product_raycell_mk2.html) would be necessary to do the same work.

X-ray irradiators have several economic and technical advantages, which include, lower capital cost, much lower transportation costs, and less stringent regulations regarding required infrastructure and safety of staff. According to Hendrichs, the average transport costs of a radioisotope source is US$50,000, which is 10 times the cost of shipping an X-ray irradiator. Regarding environmental safety, X-rays are emitted only when an x-ray machine is turned on compared to gamma-rays that are continuously emitting radioactive materials. In addition, the lower energy delivered by X-rays compared to gamma-rays requires less self-shielding, so X-ray irradiators are lighter than radionuclide irradiators.

In conclusion, this study showed that the results obtained with X-ray sterilization are quite comparable to those obtained with gamma-ray sterilization. The same dose of 110 Gy is optimal as an effectively induced sterility, counterbalancing the conservation of male biological parameters. Our findings showed that X-ray irradiators are suitable to be used in SIT programmes especially using *G. p. gambiensis*. Extending the same evaluation to other species will allow decision making about the exclusive use of X-ray technology in tsetse SIT programmes. Pending the adoption of X-ray irradiators, centres currently using gamma irradiators could test different irradiation conditions such as dose fractionation or hypoxic irradiation in order to continuously improve the quality of sterile males.

## Methods

### Biological material selection

*G. p. gambiensis* experimental flies were obtained from a colony maintained at the Insect Pest Control Laboratory (IPCL), FAO/IAEA Centre of Nuclear Techniques in Food and Agriculture, Seibersdorf, Austria. The colony was established at the IPCL in 2009 from pupae obtained from the colony of the “Centre International de Recherche-Développement sur l’Elevage en zone Subhumide” (CIRDES) in Burkina Faso. Originally, this strain was colonized at Maisons-Alfort, France in 1972 using pupae collected in Guinguette, Burkina Faso and transferred to CIRDES in 1975^61^. Additional wild material collected at the Mare aux Hippopotames was introduced into the colony in 1981.

The colony (pupae and adults) was maintained at a constant temperature of 24 ± 0.5 °C, a relative humidity (RH) of 75–80%, under subdued/indirect illumination, and with a 12 h light/12 h dark photoperiod^66,67^. Similar to the colony flies, the experimental flies were offered a blood meal three times per week on defibrinated bovine blood using an artificial membrane feeding system^66^, except the flies subjected to the survival under starvation test.

Pupae produced in the colony were collected daily and sorted by sex with a newly developed Near Infrared Pupae Sex Sorter (NIRPSS), preconditioned with the melanisation parameters set at T1 of 252, T2 of 0.10 and T3 of 10, 23–24 days post larviposition^68^. Male pupae were selected from the pupae classed as unmelanized when the unmelanized ratio (unmelanized pupae/total pupae sorted) was below 38%. Throughout all the experiments, we selected pupae that were 23–24 days old, and the total number of pupae irradiated depended on the specific type of experiment, with detailed information provided in each experiment description. The irradiation time was determined based on the doses and the dose rate of the irradiators, as described in the “Irradiation facilities and procedures” section.

### Irradiation facilities and procedures

The current study compared the gamma-ray irradiator, the Foss Model 812 and the X-ray irradiators, i.e., the Rad Source 2400 irradiator (Rad Source technology Inc., Buford, GA) and the Raycell Mk2 irradiator (Best Theratronics Ltd., Canada). Characterization and dose mapping of the two machines showed that both are suitable for the sterilization of male insects and hence, for application in SIT programmes^24,27,28^.

#### Foss Model 812 gamma irradiator

The samples were exposed to gamma-rays in normoxia in a ^60^Co Foss Model 812 with a dose rate between 76.4 and 68.9 Gy/min from the beginning to the end of the experiments. The irradiation set up was made by using the middle position of the radiation chamber on the turntable 3, the three sources A, B, and C being activated. The pupae were placed in a plastic vial (25 mm (⌀) × 50 mm (H)) with an aerated upper part which was inserted in a Plexiglas tube on a Plexiglas support in a metal canister (250 mm high and a diameter of 190 mm). The canister and the samples were placed in the middle position of the irradiation chamber, to receive the central dose.

#### Rad Source 2400 irradiator

This self-shielded low energy X-ray irradiator contains five horizontal cylindrical canisters that rotate around an X-ray tube and has an operating voltage of 150 keV, an operating current of 45 mA, a dose rate of 14.1 ± 0.7 Gy/min and a dose uniformity of 1.3^69^. The radiation canister was a cylinder (178 mm in diameter by 167 mm in length) with a squared space delimited in the centre containing a 100 × 100 × 100 mm Plexiglas to hold a Petri dish containing the pupae during irradiation^28^. The surrounding space was filled with rice, the density of which is close to that of the pupae, to follow the protocol that was used for the dose rate measurements. The pupae were placed in a small Petri dish (60 mm × 13 mm) and glued to a bigger one (90 mm × 15 mm) vertically between the Plexiglas in the centre of the square space.

#### Raycell Mk2 irradiator

This irradiator has two X-ray tubes that are located opposite from each other and that have an operating low-voltage of 160 keV with a shielded chamber of irradiation and a separate heat exchanger. The target dose was controlled by setting and monitoring the irradiation time, based on the central dose rate that was 8.23 Gy/min. The cylindrical canister had a volume of 2.0 L with a diameter 167 mm and a height of 97 mm with a central position. The irradiation was done under the same conditions as described above for the Rad Source 2400.

In all irradiators, the samples were placed in the centre of the canisters and the irradiation temperature was measured before and after each irradiation by using an RS PRO thermometer (RS Components Ltd., Northants, UK).

### Dosimetry monitoring

The quality of insect sterilisation is of paramount importance for the successful application of the SIT. Considering the different irradiators used for irradiation, it was essential to measure and confirm the actual radiation doses given to the samples. To ensure that the irradiation dose was actually absorbed by the samples with the intended target dose, an accurate and reliable dosimetry system was needed.

The dosimetry system used Gafchromic™ type HD-V2 (#lot 02202001) radiochromic dosimetry films (International Specialty Products, NJ, USA) following standard operating procedures^70^. These films have an appropriate dose–response for X- and gamma-rays irradiation^24^. Three 1 × 1 cm Gafchromic dosimetry films, individually enclosed in paper envelops, were included, and irradiated with each batch. The change in colour, as measured with a DoseReader 4 (Radiation General Ltd, Hungary), in reaction to the radiation dose over time, indicated the absorbed dose at 24 h post irradiation by reading two wavelengths, 458 nm and 590 nm^70^. The calibration used for the Rad Source 2400 and the Raycell Mk2 had a global uncertainty of 4.3% arising from multiple factors, including the dosimetry system (1.6%), the dose rate measurement (0.6%), the calibration (0.2%), the lot-non homogeneity (1.3%), the read-out temperature (0.4%) and the temperature of the dosimeter during the irradiation. The uncertainty of the Foss Model 812 was 2.9%.

### Experiment 1: dose response of pupae to gamma- and X-rays

The dose-responses of *G. p. gambiensis* pupae exposed to 70, 90, 110 and 130 Gy of radiation generated with the Foss Model 812, the Rad Source 2400, and the Raycell Mk2 was determined. Six biological replicates were conducted, each containing an average of 60 pupae for each of the four (04) irradiated treatments per irradiator, as well as the control batch subjected to the same environmental conditions.

After irradiation, the Petri dishes containing the irradiated pupae were placed under cages (~ 126 mm (Ø); 88 mm (H)) for emergence. The female flies that emerged due to the pupae sex-sorter error were subsequently discarded. The emerged irradiated males were mated on day 7–8 post emergence, in standard colony cages (20 cm diameter) with 3–4 day-old virgin non-irradiated females from the IPCL colony at a male: female ratio of 1:1 or 1:2. After 4 days, the flies were separated under chilled conditions (4 °C), and the females were transferred to standard colony cages on individual dishes , while the males were transferred to small cages (110 mm (Ø); 45 mm (H)). Male survival was monitored daily for 90 days. Female survival and pupae production/abortions were monitored daily for 60 days. Thereafter, female flies were dissected to determine their reproductive status, i.e., presence/absence of egg/larvae in the uterus and spermathecae fill level. The pupae produced were allowed to emerge and their sex was recorded. Pupae that did not emerge were dissected to determine their development stage.

Female fecundity was expressed as the number of pupae produced per mature female day as calculated for each treatment by adding the number of flies alive each day, starting on day 18 after emergence, until the end of the experiment on day 60. Induced sterility was determined by calculating the pupae production of the treatments as a proportion of the production of pupae of the control (non-irradiated ) group (100%).

### Experiment 2: flight quality control: emergence rate, flight propensity and survival

The effects of irradiation as compared with the control group on adult emergence, flight propensity and male survival under feeding stress were evaluated. The dose of 110 Gy was selected for this evaluation as it induced at least 95% sterility in the dose–response evaluation. Evaluations were conducted with fifty pupae aged 23–24-day-old for each of the control group and the three irradiators. The pupae were placed in small Petri dishes (60 mm × 13 mm) that were placed within 90 mm × 15 mm Petri dishes. A black cylinder (of 100 mm height, 94 mm inner diameter and 3 mm thickness), with the interior walls coated with talcum powder to prevent the flies from crawling out, was placed over the Petri dish containing the pupae^71^. This experiment was conducted under standard colony conditions.

Emergence from the cylinder was monitored daily and the number of flyers (i.e., flies that succeeded to fly out of the tube) were sex-sorted and recorded after chilling them in a cold room at 4 °C. Before transferring the flyers in the emergence cages to the cold room, the top of the cylinder was closed with a Petri dish to prevent the flyers from re-entering the tube. The non-flyers, remaining in the tube were also removed and recorded. The daily mortality (except for weekends) of the flyers was monitored under starvation conditions until the last fly had died. Then biological replicates have been done.

### Experiment 3: male mating performance

In SIT programmes, the mating performance of irradiated males is defined as their ability to compete with wild males to mate with wild females. To compare the effect of gamma- and X-ray irradiators on male mating performance against non-irradiated males, 23–24-days-old pupae, collected over 24 h, were sorted with the NIRPSS and exposed to 110 Gy of gamma-rays (Foss Model 812) or X-rays (Rad Source 2400). Among the two X-ray irradiators assessed in the previous experiments, the Rad Source 2400 was chosen to obtain the sex-ratio requirement of 3 males to 1 female for the mating competitiveness experiment. This decision was made due to its availability at certain SIT facilities, including the Centre International de Recherche-Developpement de l’Elevage en zone Subhumide. Considering the female contamination rate and the mortalities prior to the test date, a minimum of 50 pupae were selected for the aforementioned two treatments, as well as for the control group.

The irradiated pupae were kept in emergence cages, and the males that emerged were randomly marked with a small dot of blue, yellow, or red acrylic paint on the thoracic tergum using a wooden toothpick. Flies were offered a blood meal as described above, with the exception of the last feeding. The last feeding was divided into two parts, each lasting5 minutes, occurring two days and one day before the field cage test.

The mating experiments were carried out in a cylindrical walk-in field cage of 2.9 m in diameter and 2.0 m high. The cage is made of cream polyester netting with a flat floor and ceiling. The cages were deployed in the ecosphere of the IPCL, where a field environment can be simulated and temperature and humidity conditions can be set between 21 and 27 °C and 48 and 88% RH, respectively. A bergamot orange tree of ~ 2 m high, *Citrus aurantium,* was placed in the centre of the cage. Temperature and relative humidity were recorded every 15 min throughout the experiment with an RS PRO RS-172 Temperature and Humidity Data Logger (RS Components Ltd., UK) with an accuracy of ± 1 °C and ± 3.5% for temperature and relative humidity, respectively. Light intensity at the top of the cage, the bottom of the cage, and at the tree mid/foliage level was recorded every 15 min using a dual display light meter (VWR International, LLC, USA) with an accuracy of ± 4.5%. All experiments were carried out between 10:00 h and 15:00 h.

Thirty virgin non-irradiated 3–4-day-old females were released in the middle of the cage five minutes before the release of thirty 8–9-day-old males from each group, i.e., non-irradiated males and two groups of males irradiated with the Foss Model 812 and the Rad Source 2400. Non-irradiated and irradiated males were released simultaneously to compete for mating with non-irradiated females at a 3:1 male: female ratio, i.e., 90 males and 30 females.

The observer remained inside the cage for the 3-h duration of the experiment, keeping her/his movements to a minimum. The time when each mating couple occured was recorded, as well as the location and light intensity at that location. Mating couples were collected individually into small vials and surveyed until their separation, while also recording their duration of mating. After 3 h, all the remaining flies in the cage were collected, and the unmated females were kept separately at – 5 °C and dissected to confirm their virginity. The unmated males were discarded. Mated females were removed at the end of the mating and dissected in a phosphate buffered saline solution to assess the spermathecae filling score^72^. The experiment was repeated thirteen times.

The mating indices used were Mating Latency (ML), Mating Duration (MD), Propensity of Mating (PM), Relative Mating Index (RMI), and Relative Mating Performance (RMP)^32,61,73^. The ML was defined as the time from the end of male release to the time when the first couple is successfully formed, and MD was defined as the difference between starting time and the end of mating. The PM was defined as the pairs collected during a test as a proportion of the total possible pairs, whereas the RMI was considered as the number of males of each treatment mated as a proportion of the total number of males that mated. The RMP was determined as the difference between the number of couples of irradiated to sterile males as a proportion of the total number of couples.

### Data analysis

Data analysis was done in RStudio (RStudio, Inc. Boston, MA, United States, 2016) using the R software version 4.1.2. through different fitted models and controlled for the overdispersion.

To analyse some of the data from the dose–response experiment, Generalized Linear Mixed Models (GLMM) under the package lme4^74^ were used with the relevant family after the model overdispersion verification^75^. Thus, adult emergence and insemination rate from the experiment 3 were analysed using this model with binomial family, where the irradiators nested in the radiation type and doses were considered as the fixed effect and the replications as random effect. The same model was used with Poisson family to analyse the effect of irradiators and doses on the number of aborted eggs. To analyse spermathecae fill a GLMM with gaussian family was used after Tukey’s Ladder of Powers transformation of data.

To analyse the effect of gamma- and X-ray irradiators on male sterility/residual fertility, the dose response model with “drm” function was used under the Dose–Response Curves (drc) package^76^. The best model was selected with the “mselect” function based on the log likelihood value, Akaike's Information Criterion (AIC), known as the estimated residual standard error or the p-value from a lack-of-fit test as criteria.

The best model for the fecundity data was the Weibull three-parameters type 1 model (W1.3) given by the expression y(x) = 0 + (d−0) exp(− exp(b(log(x) − e))), while the induced sterility fitted with the Weibull model with two parameters (W2.2) expressed by y(x) = exp(− exp(b(log(x) − e))). The first model assumes that the lower limit is 0 (fecundity tended to zero at high doses), and *d* represents the upper limit of the fecundity, while *b* assigns the slope, *e* denotes the median effective irradiation dose (ED_50_) and *x* is the absorbed radiation dose (Gy). In the second model, the lower limit was fixed at 0, and the upper limit *d* is fixed at 1, being the full sterility of 100%. The curves parameters were then compared between the irradiators using the compParm function. The estimated effective doses that reduce 50, 95 and 99% of the fertility and induce the same levels of sterility for the three irradiators were determined with the ED function and then compared using the Kruskal–Wallis test.

To analyse the survival time, the Cox Mixed Effects Models (“survival” package, “coxme” function) fit by maximum likelihood was used. In this analysis, the survival time served as the response variable, and the treatments (irradiation with three different irradiators as well as a non-irradiated control group) and doses were included as fixed effects and the replications as random effect. Multiple comparisons were done using the estimated marginal means (“emmeans” function in package “emmeans”) with the Tukey p-value adjustment method. The survival graphs were constructed using “ggsurvplot” with “survimer”, “ggplot2,” and “ggpubr” packages.

For the flight quality control and the mating performance, the data were analysed using GLMM with binomial family and the overdispersion test. Adult emergence rate and flight propensity were modelled considering the treatments (irradiated with three irradiators and non-irradiated) and doses as fixed effects and replications as random effect. Male survival was analysed using the Cox Mixed Effects Model fit by maximum likelihood as in the dose response section.

The mating latency, duration and the spermathecae fill data were analysed using a Generalized Linear Model with gaussian family after Tukey’s Ladder of Powers transformation of data. The effect of the treatments as irradiated and non-irradiated and doses on the mating index was analysed with a Poisson family. During the data analysis, multiple comparisons were done using the estimated marginal means where a significant difference was found at the global level.

### Supplementary Information

Supplementary Information.

## Data Availability

The datasets used and analysed during the current study are available from the corresponding author on reasonable request.
