# Supplementary material for: X-rays are as effective as gamma-rays for the sterilization of Glossina palpalis gambiensis Vanderplank, 1911 (Diptera: Glossinidae) for use in the sterile insect technique
Source: Sci Rep. 2023 Oct 17;13:17633. doi: 10.1038/s41598-023-44479-8 (PMC10582188; doi:10.1038/s41598-023-44479-8)
Supplement: Supplementary file 1 — Supplementary Information. [file 41598_2023_44479_MOESM1_ESM.docx]

***Supplementary material***

**Experiment 1:**

1. **Supplementary Figures**

**
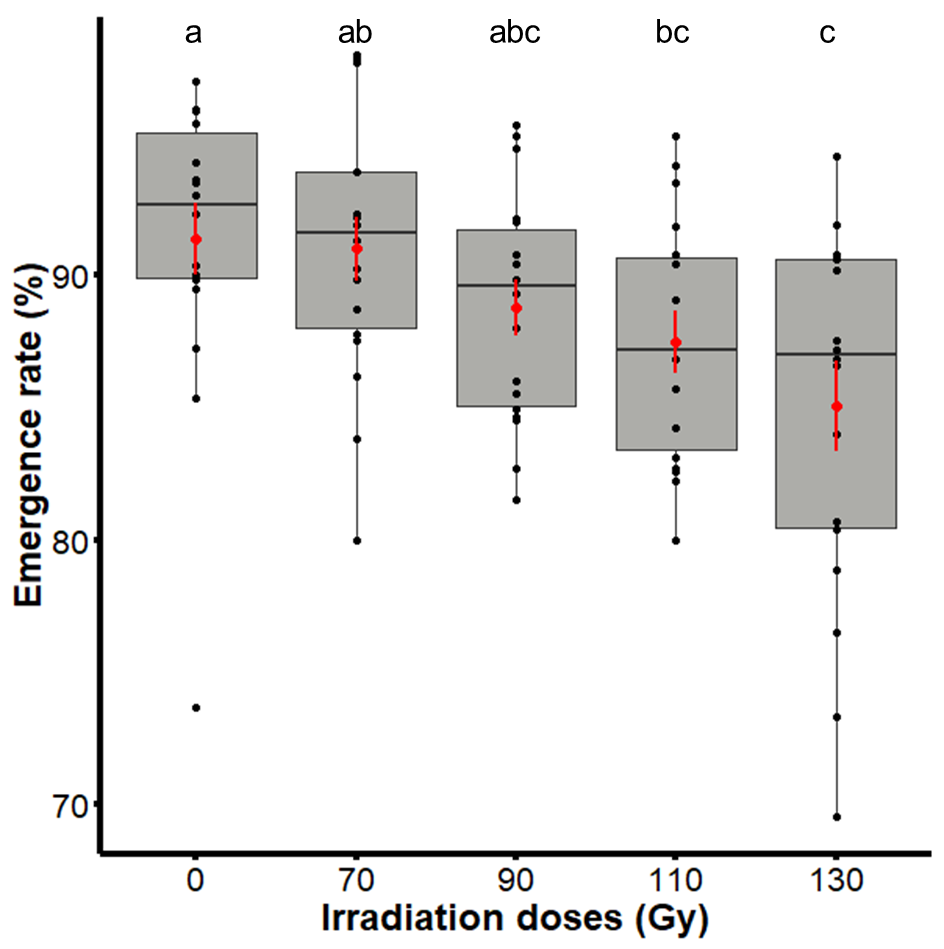
**

**Figure S1.** Adult emergence rate according to the radiation doses. The boxplots show the medians and quartiles while the means and the standard errors are highlighted in red. Different letters indicate a significant difference between the radiation doses.


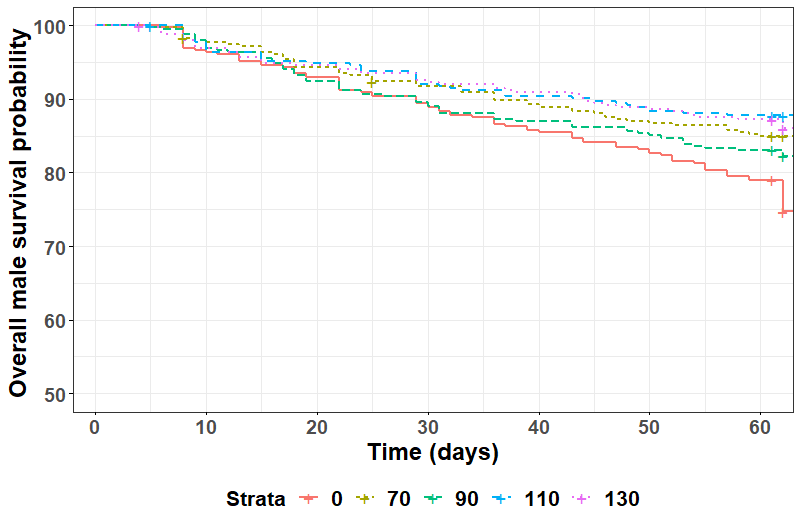


**Figure S2.** Survival curves of females mated with non-irradiated males *vs.* females mated with males irradiated with 70, 90, 110 and 130 Gy. After the 60-day experiment time, the remaining flies were dissected.


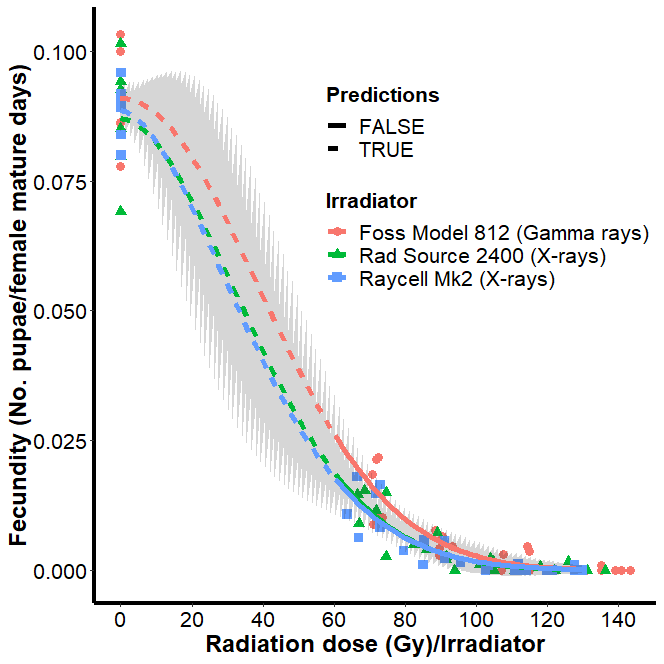


**Figure S3.** Weibull dose–response curves with 95% confidence intervals for fecundity in *Glossina palpalis gambiensis* pupae irradiated with Foss Model 812 (gamma rays), Rad Source 2400 (X-rays) and Raycell Mk2 (X-rays). The dashed lines indicate the predictive irradiation doses and sterility (Predictions = TRUE) while the solid lines and the markers represent the experiment data (Predictions = FALSE).

**Experiment 2:**


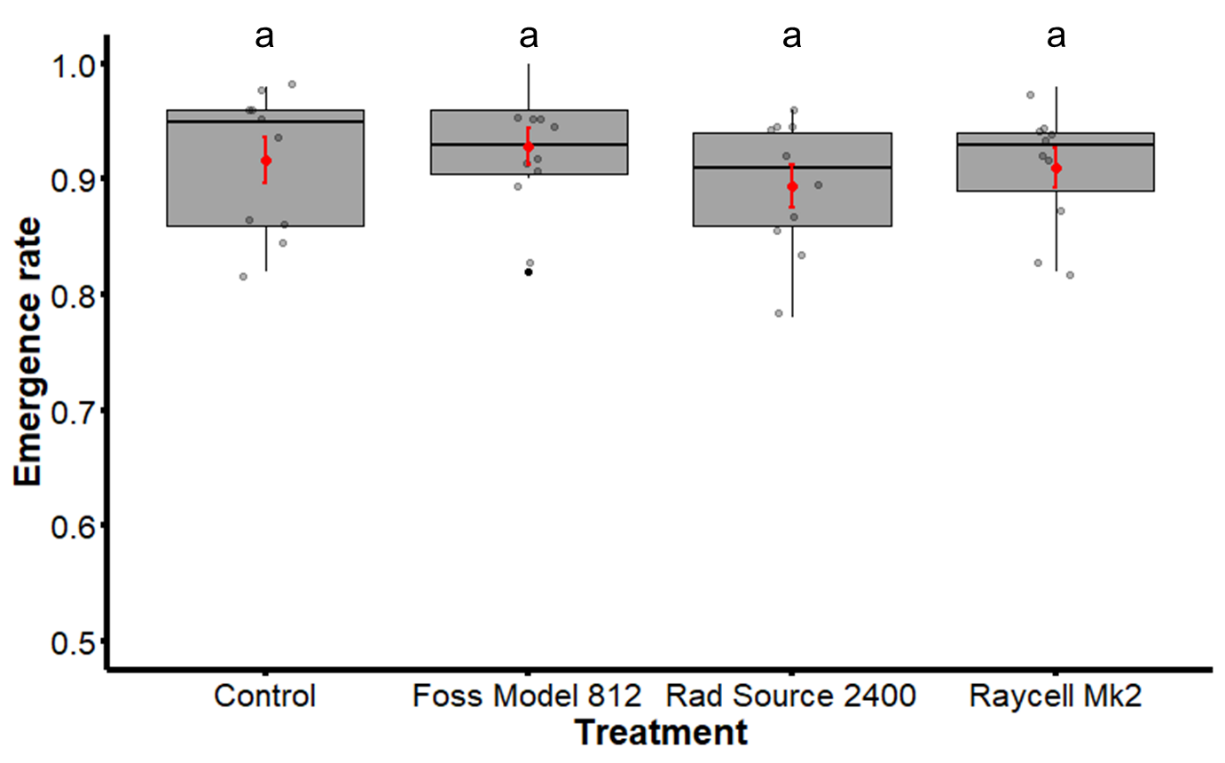


**Figure S4.** Emergence rate of *Glossina palpalis gambiensis* pupae irradiated with Foss Model 812 (Gamma rays), Rad Source 2400 (X-rays) and Raycell Mk2 (X-rays) with 110 Gy compared to non-irradiated group (Control). The boxplot shows the median, and upper and lower quartiles while the means and the standard errors are highlighted in red. There was no significant difference between the treatments.

**Experiment 3:**


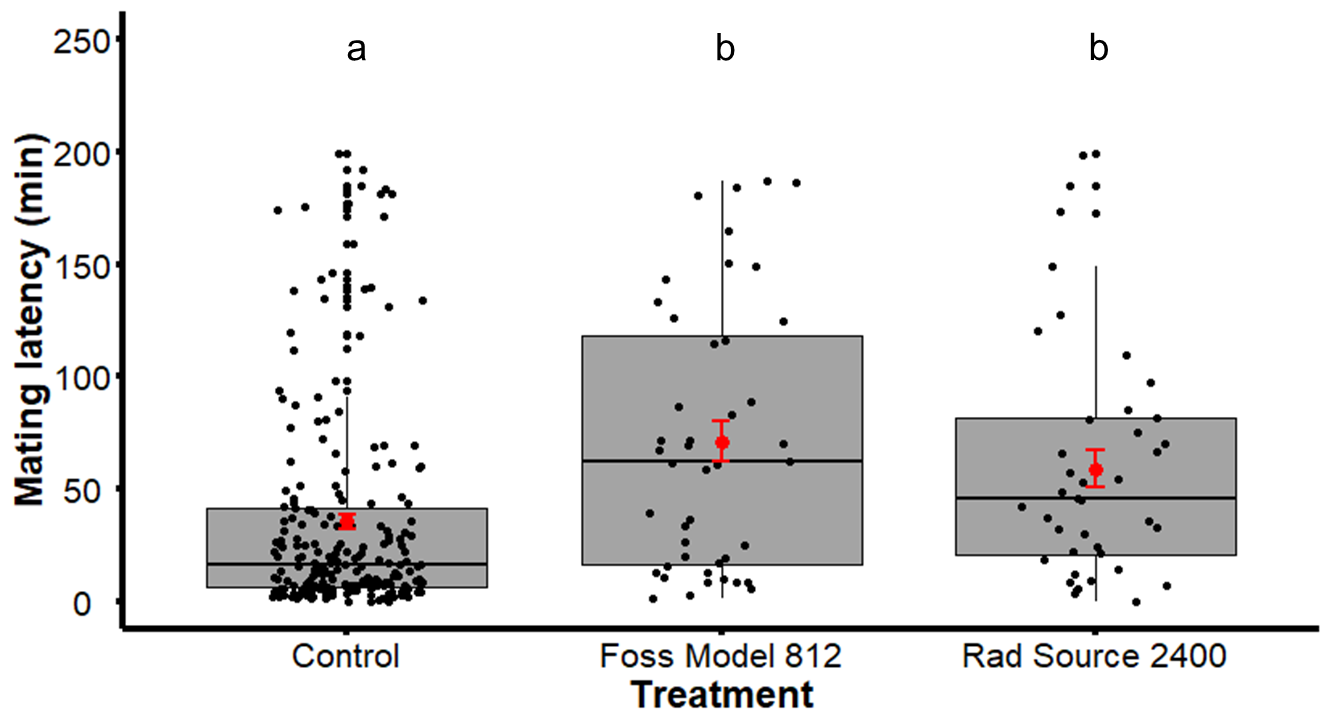


**Figure S5.** Mating latency of males irradiated with Foss model 812 (Gamma rays), and Rad Source 2400 (X-rays) compared to the non-irradiated males (Control). The boxplot shows the median, and upper and lower quartiles while the means and the standard errors are highlighted in red. Different letters indicate a significant difference between the treatments.


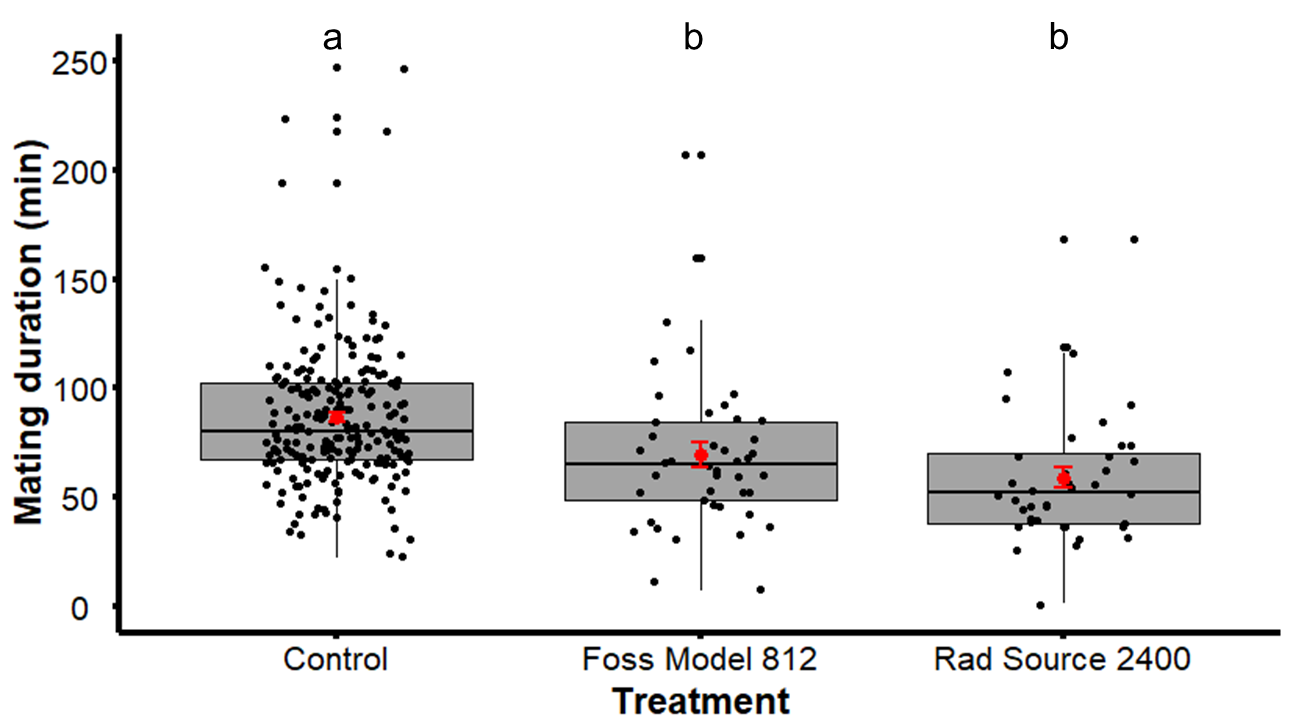


**Figure S6.** Mating duration of males irradiated with Foss Model 812 (Gamma rays) and Rad Source 2400 (X-rays) compared to the non-irradiated males. The boxplot shows the median, and upper and lower quartiles while the means and the standard errors are highlighted in red. Different letters indicate a significant difference between the treatments.

1. **Supplementary Tables**

**Table S1.** Dosimetry data regarding the range of doses used during the study. All the absorbed doses remained within the 5% variation except the dose of 130 Gy.

| Irradiator | Target dose | Mean absorbed dose | Standard deviation | Variation (%) |
| --- | --- | --- | --- | --- |
| Foss Model 812 | 70 | 72.06 | 1.00 | +2.94 |
|  | 90 | 90.61 | 1.66 | +0.68 |
|  | 110 | 112.17 | 3.82 | +1.98 |
|  | 130 | 137.55 | 4.46 | +5.81 |
| Raycell Mk2 | 70 | 69.22 | 3.92 | -1.11 |
|  | 90 | 88.07 | 5.75 | -2.15 |
|  | 110 | 109.36 | 4.87 | -0.58 |
|  | 130 | 123.55 | 6.04 | -4.96 |
| Rad Source 2400 | 70 | 70.57 | 3.98 | +0.82 |
|  | 90 | 89.14 | 7.62 | -0.96 |
|  | 110 | 106.19 | 3.62 | -3.46 |
|  | 130 | 128.36 | 4.23 | -1.26 |

**Experiment 1:**

**Table S2**. Comparison of the fecundity curves parameters between the irradiators. The curves equations are expressed as y(x) = 0 + (d − 0) exp(− exp(b(log(x) − e))) where the lower limit is fixed at 0, *d* is the upper limit, *b* is the slope and *e* is the effective dose.

| **Curves parameters** | **Compared irradiators** | **Estimate** | **Std. Error** | **t-value** | **p-value** |
| --- | --- | --- | --- | --- | --- |
| *d* | Foss Model 812-Rad Source 2400 | 0.004 | 0.003 | 1.419 | 0.160 |
|  | Foss Model 812-Raycell Mk2 | 0.002 | 0.003 | 0.868 | 0.388 |
|  | Rad Source 2400-Raycell Mk2 | -0.002 | 0.003 | -0.551 | 0.583 |
| *b* | Foss Model 812-Rad Source 2400 | 0.180 | 0.822 | 0.219 | 0.828 |
|  | Foss Model 812-Raycell Mk2 | 0.277 | 0.770 | 0.360 | 0.720 |
|  | Rad Source 2400-Raycell Mk2 | 0.098 | 0.776 | 0.126 | 0.900 |
| *e* | Foss Model 812-Rad Source 2400 | 6.295 | 8.980 | 0.701 | 0.485 |
|  | Foss Model 812-Raycell Mk2 | 8.106 | 8.638 | 0.938 | 0.351 |
|  | Rad Source 2400-Raycell Mk2 | 1.812 | 9.599 | 0.189 | 0.851 |

**Table S3.** Reproduction parameters of *Glossina palpalis gambiensis* females mated with males irradiated with gamma- and X-rays at different doses. Respectively 488, 494 and 494 females mated with males irradiated with the Foss Model 812 (Gamma rays), Rad Source 2400 (X-rays) and Raycell Mk2 (X-rays) were dissected on day 60. Fecundity is expressed as the number of pupae produced per mature female days. Mature female days were calculated for each treatment by adding the number of flies alive each day, starting on day 18 after emergence (age of maturity) until the end of the experiment on day 60.

| **Irradiators and doses (Gy)** | **Pupae emergence (%)** | **Mature female days** | **No of aborted eggs** | **No of pupae produced** | **Fecundity** | **Induced sterility (%)** | **F1 pupae emergence rate / Percentage of females (%)** | **Age of F1 pupae at emergence (days)** |
| --- | --- | --- | --- | --- | --- | --- | --- | --- |
| Foss Model 812 |  |  |  |  |  |  |  |  |
| 0 | 93.2±2.9 | 4501 | 15 | 402 | 0.091 | 0 | 95.6/49.2 | 33±0.8 |
| 70 | 92.2±3.6 | 4724 | 435 | 76 | 0.015 | 82.2±7.1 | 88.1/51.7 | 33±1.0 |
| 90 | 88.0±4.9 | 4610 | 519 | 23 | 0.005 | 94.7±2.0 | 75.0/47.2 | 33±1.3 |
| 110 | 89.6±5.4 | 4850 | 540 | 11 | 0.002 | 97.7±2.7 | (8/11)/(3/8) | 33±1.3 |
| 130 | 85.7±4.9 | 4863 | 490 | 1 | <0.001 | 99.8±0.6 | (1/1)/(1/1) | 32* |
| Rad Source 2400 |  |  |  |  |  |  |  |  |
| 0 | 91.4±10.0 | 4597 | 42 | 406 | 0.087 | 0 | 96.3/48.8 | 33±0.7 |
| 70 | 91.5±6.8 | 4811 | 480 | 59 | 0.011 | 86.4±6.3 | 84.5/60.2 | 32±1.1 |
| 90 | 87.4±5.0 | 4603 | 529 | 15 | 0.003 | 95.9±2.5 | (15/15)/(4/15) | 34±1.0 |
| 110 | 88.9±3.4 | 4969 | 564 | 5 | <0.001 | 98.8±1.4 | (5/5)/(3/5) | 31±2.1 |
| 130 | 85.7±9.2 | 4906 | 519 | 2 | <0.001 | 99.4±0.9 | (1/2)/(0/2) | 36* |
| Raycell Mk2 |  |  |  |  |  |  |  |  |
| 0 | 89.7±2.9 | 4602 | 31 | 406 | 0.088 | 0 | 95.7/51.5 | 33±0.6 |
| 70 | 89.2±4.1 | 4777 | 458 | 53 | 0.012 | 84.9±6.7 | 83.9/29.3 | 33±1.0 |
| 90 | 90.9±2.6 | 4864 | 531 | 15 | 0.003 | 95.7±3.6 | 95.8/42.2 | 33±1.5 |
| 110 | 83.9±2.9 | 4731 | 515 | 2 | <0.001 | 99.6±0.6 | (2/2)/(0/2) | 37±2.1 |
| 130 | 83.8±7.4 | 4761 | 499 | 1 | <0.001 | 99.8±0.5 | (1/1)/(0/1) | 35* |

*Only one pupae were produced or emerged from these treatments

**Table S4.** Reproductive status of *Glossina palpalis gambiensis* females, that mated with males irradiated with Foss Model 812 (Gamma rays), Rad Source 2400 (X-rays) and Raycell Mk2 (X-rays) at different doses, dissected after an experimental period of 60 days.

| **Irradiators and doses (Gy)** | **No of females alive at day 60** | **Insemination rate** | **Spermathecae fill score** | | | | | **Uterus content or status at day 60** | | | | | |
| --- | --- | --- | --- | --- | --- | --- | --- | --- | --- | --- | --- | --- | --- |
|  |  |  |  |  |  |  |  | **No of recently ovulated eggs** | **Viable instar larvae** | | | **Empty due to** | |
|  |  |  | **0** | **0.25** | **0.5** | **0.75** | **1** |  | **I** | **II** | **III** | **Larviposition** | **Abortion** |
| Foss Model 812 |  |  |  |  |  |  |  |  |  |  |  |  |  |
| 0 | 101 | 1.00 | 0 | 20 | 26 | 40 | 0 | 17 | 16 | 10 | 16 | 22 | 5 |
| 70 | 89 | 0.98 | 2 | 14 | 33 | 45 | 6 | 20 | 0 | 1 | 7 | 5 | 67 |
| 90 | 101 | 0.98 | 1 | 14 | 27 | 49 | 2 | 14 | 1 | 1 | 1 | 0 | 76 |
| 110 | 94 | 0.95 | 7 | 11 | 29 | 47 | 5 | 18 | 0 | 1 | 0 | 4 | 76 |
| 130 | 103 | 0.95 | 11 | 24 | 31 | 31 | 5 | 27 | 5 | 1 | 0 | 2 | 67 |
| Rad Source 2400 |  |  |  |  |  |  |  |  |  |  |  |  |  |
| 0 | 91 | 1.00 | 0 | 26 | 23 | 30 | 11 | 26 | 20 | 4 | 25 | 14 | 1 |
| 70 | 98 | 0.91 | 5 | 20 | 30 | 34 | 8 | 31 | 1 | 2 | 4 | 2 | 58 |
| 90 | 97 | 0.99 | 3 | 11 | 27 | 49 | 9 | 26 | 1 | 0 | 0 | 2 | 70 |
| 110 | 106 | 0.96 | 7 | 19 | 27 | 44 | 10 | 12 | 0 | 0 | 1 | 4 | 84 |
| 130 | 102 | 0.97 | 9 | 35 | 35 | 17 | 2 | 17 | 0 | 0 | 0 | 0 | 81 |
| Raycell Mk2 |  |  |  |  |  |  |  |  |  |  |  |  |  |
| 0 | 89 | 0.99 | 2 | 6 | 17 | 60 | 4 | 21 | 13 | 11 | 18 | 23 | 3 |
| 70 | 100 | 0.95 | 4 | 5 | 27 | 55 | 8 | 20 | 5 | 3 | 5 | 1 | 65 |
| 90 | 103 | 0.99 | 1 | 23 | 34 | 38 | 3 | 13 | 4 | 0 | 0 | 1 | 81 |
| 110 | 101 | 0.96 | 6 | 21 | 34 | 38 | 0 | 8 | 2 | 0 | 2 | 2 | 85 |
| 130 | 101 | 0.94 | 6 | 13 | 30 | 55 | 1 | 20 | 3 | 0 | 0 | 3 | 79 |

**Table S5.** Comparison of the induced sterility curves parameters between the irradiators. The curves equations are expressed as y(x) = exp(−exp(b(log(x)−e))) where the lower limit was fixed at 0, *d* the upper limit was fixed at 1, *b* is the slope and *e* is the effective dose.

| **Curves parameters** | **Compared irradiators** | **Estimate** | **Std. Error** | **t-value** | **p-value** |
| --- | --- | --- | --- | --- | --- |
| *b* | Raycell Mk2-Foss Model 812 | -0.314627 | 0.458464 | -0.6863 | 0.4944 |
|  | Raycell Mk2-Rad Source 2400 | -0.287102 | 0.49334 | -0.582 | 0.5622 |
|  | Foss Model 812-Rad Source 2400 | 0.027525 | 0.53506 | 0.0514 | 0.9591 |
| *e* | Raycell Mk2-Foss Model 812 | -7.8131 | 4.4593 | -1.7521 | 0.08341 |
|  | Raycell Mk2-Rad Source 2400 | -2.9492 | 4.8609 | -0.6067 | 0.54567 |
|  | Foss Model 812-Rad Source 2400 | 4.8638 | 4.6379 | 1.0487 | 0.29732 |

**Experiment 2:**

**Table S6.** Statistics of the pairwise comparisons of the male survival time under feeding stress condition using estimated marginal means

| **Treatment** | **Raycell Mk2** | **Rad Source 2400** | **Foss Model 812** |
| --- | --- | --- | --- |
| Rad Source 2400 | 0.886 | - | - |
| Foss Model 812 | 0.056 | 0.055 | - |
| Control | 8.50E-07 | 3.30E-05 | 1.40E-12 |

**Experiment 3:**

**Table S7.** Average distribution of locations and lux intensity at which mating pairs were collected in the field cage, after competition between fertile males and males irradiated with Foss Model 812 and Rad Source 2400

| Location in the cage | No of mating pairs (N = 297) | Frequency (%) | | | Lux intensity (Lx) (Mean±SD) |
| --- | --- | --- | --- | --- | --- |
|  |  | **Control** | **Foss Model 812** | **Rad Source 2400** |  |
| Bottom of the cage | 25 | 68.0 | 20.0 | 12.0 | 567.76±252.09 |
| Middle of the cage (wall) | 27 | 63.0 | 18.5 | 18.5 | 864.00±405.83 |
| On the tree | 2 | 0.0 | 50.0 | 50.0 | 1004.50±1042.98 |
| Top of the cage | 243 | 73.7 | 13.6 | 12.8 | 1239.83±551.18 |

**Table S8.** Statistical analysis of spermathecal fill. Treatment and mating duration were considered as explanatory variables.

|  | LR Chisq | Df | Pr(>Chisq) |
| --- | --- | --- | --- |
| Treatment | 7.3139 | 2 | 0.02581 |
| Duration | 1.1769 | 1 | 0.27799 |
| Treatment*duration | 4.656 | 2 | 0.09749 |

**Table S9.** Pairwise comparison of the spermathecae fill between the treatments. There was no significant difference.

| Treatments | estimate | SE | df | t.ratio | p.value |
| --- | --- | --- | --- | --- | --- |
| Control – Foss Model 812 | 0.06818 | 0.0503 | 291 | 1.355 | 0.366 |
| Control – Rad Source 2400 | 0.07599 | 0.06 | 291 | 1.266 | 0.4154 |
| Foss Model 812 – Rad Source 2400 | 0.00781 | 0.0728 | 291 | 0.107 | 0.9937 |
